# Role of Opioid-Sparing Techniques in Pain Management for General Surgery Patients With Hepatic Dysfunction: An Observational Cohort Study

**DOI:** 10.7759/cureus.77117

**Published:** 2025-01-07

**Authors:** Dulce M Rascón-Martínez, Samid Ullah, Muhammad Anwar Memon, Adil Bangash, Ayub Ali, Muhammad Ikram Ul Haq, Arif Mohyuddin, Muhammad Kamran Khan, Fatima Ovais, Imtiaz Mustafa

**Affiliations:** 1 Anesthesia, Instituto Mexicano del Seguro Social (IMSS), Mexico City, MEX; 2 Gastroenterology and Hepatology, Khalifa Gul Nawaz Teaching Hospital, Medical Teaching Institution Bannu, Bannu, PAK; 3 General Surgery, Liaquat University of Medical and Health Sciences, Jamshoro, PAK; 4 General Surgery, Lady Reading Hospital, Peshawar, PAK; 5 College of Medicine, King Faisal University, Hofuf, SAU; 6 Medicine, Niazi Medical and Dental College, Sargodha, PAK; 7 Biomedical Sciences, King Faisal University, Hofuf, SAU; 8 Medicine, College of Medicine - Dawadmi, Shaqra University, Riyadh, SAU; 9 Anesthesia, Dr. Faisal Masood Teaching Hospital, Sargodha, PAK; 10 Physiology, The University of Lahore, Lahore, PAK

**Keywords:** general surgery, hepatic dysfunction, opioid-sparing techniques, pain management, postoperative outcomes, regional anesthesia

## Abstract

Introduction

Patients with hepatic dysfunction undergoing general surgery face significant perioperative risks due to the liver’s critical role in drug metabolism and systemic homeostasis. Opioid-sparing techniques may mitigate these risks by reducing opioid consumption and minimizing hepatic complications.

Objective

The objective of the study was to evaluate the effectiveness of opioid-sparing techniques in pain management for general surgery patients with hepatic dysfunction and their impact on postoperative outcomes.

Methodology

This observational cohort study was conducted at Lady Reading Hospital, Peshawar, Pakistan, from November 2023 to October 2024. A total of 100 patients with hepatic dysfunction undergoing elective general surgeries were included, with 50 managed using opioid-sparing techniques and 50 receiving opioid-based analgesia. Pain was assessed using the Numeric Rating Scale (NRS) at six, 12, 24, and 48 hours post-surgery. Opioid consumption was quantified as morphine-equivalent doses over 48 hours. Hepatic function parameters (aspartate aminotransferase (AST), alanine aminotransferase (ALT), and total bilirubin) and postoperative outcomes, including complications and hospital stay, were analyzed.

Results

Postoperative pain scores were significantly lower in the opioid-sparing group across all time points (e.g., NRS at six hours: 2.9 ± 0.8 vs. 4.5 ± 1.1, p < 0.001). Opioid consumption was reduced by 67% in the opioid-sparing group (4.1 ± 1.5 mg vs. 12.6 ± 3.8 mg,p < 0.001). Hepatic function parameters remained stable in both groups. The opioid-sparing group had shorter hospital stays (3.8 ± 0.9 days vs. 5.1 ± 1.2 days, p < 0.001) and fewer complications, including nausea (12% vs. 30%, p = 0.02) and respiratory depression (0% vs. 10%, p = 0.04).

Conclusion

Opioid-sparing techniques improve pain management, reduce opioid consumption, and minimize postoperative complications in patients with hepatic dysfunction undergoing general surgery. These findings support incorporating opioid-sparing protocols into perioperative care for this high-risk population.

## Introduction

The liver is the primary internal organ of the body, weighing approximately 1.4 kg in women and 1.6 kg in men. It performs several critical functions, including bile production, plasma protein synthesis, drug metabolism, and storage of vitamins, minerals, and glucose [1]. Liver disease is a multisystem disorder that can be classified as acute or chronic based on the duration between the onset of symptoms and the triggering event [2]. Acute liver failure is characterized by severe liver damage symptoms, such as encephalopathy and reduced synthetic function, in a patient with no prior history of liver disease, typically occurring within 26 weeks of illness onset [3].

Globally, liver disease remains a significant health concern, with an estimated two million deaths annually due to complications such as cirrhosis, viral hepatitis, and hepatocellular carcinoma. The prevalence and severity of liver disease are influenced by race, socioeconomic status, and geographic location [4]. Factors contributing to the rise in chronic liver disease include alcohol consumption, metabolic dysfunction-associated steatotic liver disease (MASLD), hepatitis B (HBV), and hepatitis C (HCV) [5].

Patients with liver disease, particularly those with cirrhosis, often require surgery, and anesthesia can pose substantial risks. General, spinal, or epidural anesthesia during surgery may lead to slight increases in serum liver biochemical markers, which are generally not clinically significant in individuals without underlying liver disease. However, in patients with hepatic dysfunction, surgery can accelerate hepatic decompensation and exacerbate morbidity and mortality risks. These risks are correlated with the intensity of the surgical procedure [6].

Selecting anesthetics with lower hepatotoxicity is crucial for minimizing the worsening of liver function in patients with elevated liver enzyme levels. Preoperative evaluation is essential for risk assessment and optimization before elective surgeries. Additionally, postoperative care should prioritize rehabilitation and monitoring for hepatic decompensation. Non-hepatic surgeries are more common and often performed during acute care situations [7-9].

Hepatic dysfunction resulting from chronic hepatocyte damage, cirrhosis, and portal hypertension impacts multiple organ systems and complicates surgical outcomes. Patients with hepatic dysfunction exhibit systemic vasodilation, impaired diastolic function, and insufficient responses to surgical stress [10,11]. Despite the increasing prevalence of liver disease and its significant impact on perioperative outcomes, there is a lack of locally developed, evidence-based protocols for managing these patients in surgical settings. Effective management requires a multidisciplinary approach, with attention to preoperative optimization, intraoperative anesthetic management, and postoperative rehabilitation.

This study focuses on the role of opioid-sparing techniques in pain management for general surgery patients with hepatic dysfunction. Opioid-sparing strategies, which include the use of regional anesthesia, multimodal analgesia, and non-opioid pharmacological agents, may reduce the risk of hepatic complications by minimizing the systemic effects of opioids and their impact on liver metabolism. By optimizing pain management, these techniques aim to improve perioperative outcomes and enhance recovery in this high-risk patient population.

## Materials and methods

This observational cohort study was conducted at Lady Reading Hospital, Peshawar, Pakistan, over a one-year period from November 2023 to October 2024. The study aimed to evaluate the effectiveness of opioid-sparing techniques in managing postoperative pain in patients with hepatic dysfunction undergoing general surgery. The study was approved by the Institutional Review Board, Lady Reading Hospital, Peshawar, Pakistan (approval number: 417/LRH/MTI).

Inclusion and exclusion criteria

The study included patients aged between 18 and 65 years who were diagnosed with hepatic dysfunction classified as Child-Pugh A or B [12]. Patients were selected if they were scheduled for elective general surgeries such as cholecystectomy (either laparoscopic or open), hernia repair (inguinal or ventral), appendectomy, or segmental colectomy, had an American Society of Anesthesiologists (ASA) physical status II or III, and provided informed written consent were included in the study. Patients with more severe hepatic dysfunction classified as Child-Pugh C, those requiring emergency surgeries, or those with known hypersensitivity to analgesic medications such as non-steroidal anti-inflammatory drugs (NSAIDs), acetaminophen, or opioids were excluded. Additionally, patients with significant comorbidities such as chronic kidney disease, cardiovascular disease, or neurological disorders were not included. Those undergoing surgeries with an anticipated operative time exceeding four hours were also excluded.

Sample size

The sample consisted of 100 patients, with 50 patients allocated to the opioid-sparing group and 50 to the opioid-based group based on the analgesia protocol followed during their perioperative care. Patients were assigned to these groups based on the clinical decisions of their treating physicians, without any randomization, ensuring that the study reflected real-world clinical practices.

Intervention

Patients in the opioid-sparing group received multimodal analgesia, which included intravenous acetaminophen administered at a dose of 1 gm every eight hours, ketorolac given at 30 mg every eight hours, and a continuous infusion of dexmedetomidine at a rate of 0.3-0.5 μg/kg/hour. The opioid-based group, in contrast, received conventional analgesia, primarily involving morphine administered at doses of 2-4 mg intravenously every four to six hours on an as-needed basis. Both groups had access to rescue analgesia if their pain scores exceeded 4 on the Numeric Rating Scale (NRS) [13].

Data collection

Data collection was conducted systematically using standardized forms. Pain scores were measured at regular intervals postoperatively, specifically at six, 12, 24, and 48 hours, using the NRS pain score. Opioid consumption was quantified by calculating the total morphine-equivalent dose consumed within 48 hours post surgery. To monitor hepatic function, levels of aspartate aminotransferase (AST), alanine aminotransferase (ALT), and total bilirubin were measured preoperatively and postoperatively on days 1 and 3. Adverse effects, including nausea, vomiting, respiratory depression, and pruritus, were recorded during the hospital stay. The length of hospital stays, measured in days from admission to discharge, was also documented.

Data analysis

The collected data were analyzed using IBM SPSS Statistics for Windows, Version 26.0 (Released 2019; IBM Corp., Armonk, New York, United States). Continuous variables such as pain scores, opioid consumption, and laboratory parameters were summarized as means with standard deviations (SD) and compared between the two groups using an independent t-test. Categorical variables, including the incidence of adverse effects, were expressed as frequencies and percentages and analyzed using the chi-square test. A p-value of less than 0.05 was considered statistically significant.

## Results

The demographic and baseline characteristics of patients were comparable between the two groups (Table 1). The opioid-sparing group had a mean age of 49.3 ± 8.7 years, while the opioid-based group had a mean age of 48.9 ± 9.2 years (p = 0.78). Gender distribution and BMI were also similar, with no significant differences observed. 

**Table 1:** Demographic and baseline characteristics of patients (N=100) *Independent t-test for continuous variables; **Chi-square test for categorical variables. p value less than 0.05 was considered significant

Parameter	Opioid-Sparing Group (n=50)	Opioid-Based Group (n=50)	p-value
Age (years), mean ± SD	49.3 ± 8.7*	48.9 ± 9.2	0.78
Gender (male), n (%)	28 (56%)**	29 (58%)	0.85
BMI (kg/m²), mean ± SD	27.1 ± 2.8*	27.3 ± 3.1	0.67

Postoperative pain scores assessed using the NRS were significantly lower in the opioid-sparing group at all time points (Table 2). At six hours post surgery, the opioid-sparing group had a mean NRS score of 2.9 ± 0.8 compared to 4.5 ± 1.1 in the opioid-based group (p < 0.001). This trend continued through 48 hours post surgery.

**Table 2:** Postoperative pain scores at different time points Independent t-test; p value less than 0.05 was considered significant NRS: Numeric Rating Scale

Time Point	NRS score, Opioid-Sparing Group (n=50), mean ± SD	NRS score, Opioid-Based Group (n=50), mean ± SD			p-value
6 hours post-surgery	2.9 ± 0.8	4.5 ± 1.1			<0.001
12 hours post-surgery	2.3 ± 0.7	3.8 ± 1.0			<0.001
24 hours post-surgery	1.8 ± 0.5	3.1 ± 0.9			<0.001
48 hours post-surgery	1.2 ± 0.4	2.5 ± 0.7			<0.001

Hepatic function parameters, including AST, ALT, and total bilirubin, showed no significant differences between the groups on postoperative day 3 (Table 3).

**Table 3:** Hepatic function parameters on postoperative day 3 Independent t-test; p value less than 0.05 was considered significant

Parameter	Opioid-Sparing Group (n=50), mean ± SD	Opioid-Based Group (n=50), mean ± SD	p-value
AST (IU/L)	48.7 ± 10.1	50.3 ± 9.8	0.28
ALT (IU/L)	45.6 ± 9.2	46.8 ± 10.3	0.35
Total bilirubin (mg/dL)	1.2 ± 0.3	1.3 ± 0.4	0.19

Surgical outcomes showed significant differences favoring the opioid-sparing group. The mean length of hospital stay was 3.8 ± 0.9 days in the opioid-sparing group compared to 5.1 ± 1.2 days in the opioid-based group (p < 0.001). Rates of nausea, respiratory depression, and pruritus were significantly lower in the opioid-sparing group (Table 4).

**Table 4:** Surgical outcomes *Independent t-test for continuous variables; **Chi-square test for categorical variables; p value less than 0.05 was considered significant

Outcome	Opioid-Sparing Group (n=50)	Opioid-Based Group (n=50)	p-value
Length of hospital stay (days), mean ± SD	3.8 ± 0.9	5.1 ± 1.2	<0.001*
Nausea and vomiting, n (%)	6 (12%)	15 (30%)	0.02**
Respiratory depression, n (%)	0 (0%)	5 (10%)	0.04**
Pruritus, n (%)	2 (4%)	8 (16%)	0.03**

The distribution of surgical procedures was comparable between groups, with no significant differences (Table 5).

**Table 5:** Types of surgeries performed Chi-square test for categorical variables; p value less than 0.05 was considered significant

Surgery Type	Opioid-Sparing Group (n=50), n (%)	Opioid-Based Group (n=50), n (%)	p-value
Cholecystectomy	22 (44%)	21 (42%)	0.81
Hernia Repair	16 (32%)	17 (34%)	0.77
Appendectomy	8 (16%)	7 (14%)	0.79
Segmental Colectomy	4 (8%)	5 (10%)	0.68

## Discussion

The demographic and baseline characteristics, including age, gender, and BMI, were comparable between the opioid-sparing and opioid-based groups. This ensures that the differences observed in outcomes are attributable to the analgesic strategies rather than demographic disparities. The comparability of these variables aligns with previous literature emphasizing the importance of balanced baseline characteristics in studies involving patients with hepatic dysfunction, as these factors can influence both pharmacokinetics and pharmacodynamics in the perioperative period [14,15].

Postoperative pain scores assessed using the NRS were significantly lower in the opioid-sparing group at all time points, with reductions of approximately 35-45% compared to the opioid-based group. This improvement is likely due to the multimodal analgesic approach, which combines non-opioid medications such as acetaminophen and NSAIDs with regional anesthesia techniques. These strategies provide targeted pain relief by modulating central and peripheral pain pathways without overburdening hepatic metabolism [16,17]. Acetaminophen, when administered at therapeutic doses, is relatively safe for patients with liver dysfunction, as it bypasses the pathways responsible for significant hepatotoxicity [18]. Moreover, regional anesthesia reduces systemic opioid requirements by directly blocking pain transmission, a finding supported by its efficacy in patients with chronic liver disease undergoing surgery [19].

The opioid consumption over 48 hours was significantly lower in the opioid-sparing group, with a mean morphine-equivalent dose of 4.1 ± 1.5 mg compared to 12.6 ± 3.8 mg in the opioid-based group. This reduction is critical for patients with hepatic dysfunction, as opioids undergo hepatic metabolism and may accumulate in the presence of impaired liver function, leading to adverse effects such as sedation and hepatic encephalopathy [20,21]. Dexmedetomidine, a key component of the opioid-sparing protocol, acts as a selective alpha-2 adrenergic receptor agonist, providing analgesia and sedation without significantly impacting hepatic enzymes [22]. This property makes it a preferable choice for patients with compromised liver function [11,22].

Hepatic function parameters, including AST, ALT, and total bilirubin, showed no significant differences between the two groups on postoperative day 3. This finding suggests that opioid-sparing regimens do not exacerbate liver dysfunction, likely due to the use of medications with minimal hepatotoxicity. Prior studies have highlighted the hepatoprotective effects of avoiding opioids and using non-opioid alternatives that do not interfere with liver function [11,22]. Additionally, the reduction in systemic opioid exposure may have mitigated the risk of drug-induced hepatic injury, which is a concern in cirrhotic patients [22,23].

Surgical outcomes, including the length of hospital stay and rates of postoperative complications, favored the opioid-sparing group. The mean hospital stay was shorter by approximately 1.3 days, and rates of nausea, respiratory depression, and pruritus were significantly lower. These findings align with previous studies that demonstrated improved recovery profiles and reduced opioid-related complications with multimodal analgesia [22,24]. The shorter hospital stay may also reflect better pain management, leading to enhanced mobilization and reduced risks of complications such as infections and thromboembolic events [25].

Limitations and future recommendations

This study has several limitations. First, the sample size was relatively small, limiting the generalizability of the findings. Second, the study was conducted at a single center, which may not reflect variability in practice across different institutions. Third, the follow-up period was limited to the immediate postoperative phase, precluding assessment of long-term outcomes and complications.

Future studies should include larger, multicenter cohorts to validate these findings and explore the long-term impact of opioid-sparing techniques on hepatic outcomes. Additionally, investigations into the cost-effectiveness and patient satisfaction associated with these protocols are recommended to guide broader clinical adoption [26,27]. This study underscores the importance of developing evidence-based perioperative management strategies tailored to patients with hepatic dysfunction. Incorporating opioid-sparing techniques into standard care protocols has the potential to enhance perioperative outcomes and reduce the burden of complications in this high-risk population.

## Conclusions

This study underscores the effectiveness of opioid-sparing techniques in managing postoperative pain and optimizing recovery in general surgery patients with hepatic dysfunction. These strategies significantly reduced pain scores and opioid consumption while minimizing opioid-related adverse effects and maintaining stable hepatic biomarkers, including AST, ALT, and total bilirubin. The findings highlight the potential of multimodal analgesia to enhance recovery, shorten hospital stays, and reduce complications, particularly in patients with impaired hepatic metabolism.

## References

[1] Shanmugam H, Di Ciaula A, Di Palo DM (2021). Multiplying effects of COVID-19 lockdown on metabolic risk and fatty liver. Eur J Clin Invest.

[2] Choudhury A, Rajaram R, Sarin SK (2024). Acute-on-chronic liver failure in metabolic dysfunction-associated fatty liver disease patients: a disease multiplier. Hepatol Int.

[3] Spring A, Saran JS, McCarthy S, McCluskey SA (2020). Anesthesia for the patient with severe liver failure. Anesthesiol Clin.

[4] Younossi ZM, Wong G, Anstee QM, Henry L (2023). The global burden of liver disease. Clin Gastroenterol Hepatol.

[5] Golabi P, Paik JM, Eberly K, de Avila L, Alqahtani SA, Younossi ZM (2022). Causes of death in patients with non-alcoholic fatty liver disease (NAFLD), alcoholic liver disease and chronic viral hepatitis B and C. Ann Hepatol.

[6] Hansen JD, Perri RE, Riess ML (2021). Liver and biliary disease of pregnancy and anesthetic implications: a review. Anesth Analg.

[7] Abbas N, Fallowfield J, Patch D (2023). Guidance document: risk assessment of patients with cirrhosis prior to elective non-hepatic surgery. Frontline Gastroenterol.

[8] Newman KL, Johnson KM, Cornia PB, Wu P, Itani K, Ioannou GN (2020). Perioperative evaluation and management of patients with cirrhosis: risk assessment, surgical outcomes, and future directions. Clin Gastroenterol Hepatol.

[9] Hickman L, Tanner L, Christein J, Vickers S (2019). Non-hepatic abdominal surgery in patients with cirrhotic liver disease. J Gastrointest Surg.

[10] Northup PG, Garcia-Pagan JC, Garcia-Tsao G (2021). Vascular liver disorders, portal vein thrombosis, and procedural bleeding in patients with liver disease: 2020 practice guidance by the American Association for the Study of Liver Diseases. Hepatology.

[11] Oh SK, Lim BG, Kim YS, Kim SS (2020). Comparison of the postoperative liver function between total intravenous anesthesia and inhalation anesthesia in patients with preoperatively elevated liver transaminase levels: a retrospective cohort study. Ther Clin Risk Manag.

[12] El-Khateeb E, Darwich AS, Achour B, Athwal V, Rostami-Hodjegan A (2021). Review article: time to revisit Child-Pugh score as the basis for predicting drug clearance in hepatic impairment. Aliment Pharmacol Ther.

[13] Hjermstad MJ, Fayers PM, Haugen DF (2011). Studies comparing numerical rating scales, verbal rating scales, and visual analogue scales for assessment of pain intensity in adults: a systematic literature review. J Pain Symptom Manage.

[14] Haas JT, Francque S, Staels B (2016). Pathophysiology and mechanisms of nonalcoholic fatty liver disease. Annu Rev Physiol.

[15] Muilenburg DJ, Singh A, Torzilli G, Khatri VP (2009). Surgery in the patient with liver disease. Med Clin North Am.

[16] Douard R, Lentschener C, Ozier Y, Dousset B (2009). Operative risks of digestive surgery in cirrhotic patients. Gastroenterol Clin Biol.

[17] Hernaez R, Kramer JR, Khan A (2022). Depression and anxiety are common among patients with cirrhosis. Clin Gastroenterol Hepatol.

[18] Rubin JB, Lai JC, Shui AM, Hohmann SF, Auerbach A (2021). Patterns of inpatient opioid use and related adverse events among patients with cirrhosis: a propensity‐matched analysis. Hepatol Commun.

[19] Gilbert-Kawai N, Hogan B, Milan Z (2022). Perioperative management of patients with liver disease. BJA Educ.

[20] Shahbar A, Katlan DA, Almutairy RF, Alnoamy Y (2024). Optimizing medication therapy in elderly patients: therapeutic management challenges and strategies. Difficulties and Challenges in Geriatric Health Management.

[21] Freo U, Ruocco C, Valerio A, Scagnol I, Nisoli E (2021). Paracetamol: a review of guideline recommendations. J Clin Med.

[22] Soleimanpour H, Safari S, Rahmani F, Jafari Rouhi A, Alavian SM (2015). Intravenous hypnotic regimens in patients with liver disease; a review article. Anesth Pain Med.

[23] Cheppalli NS, Metikala S, Beale J, Mounsamy V, Sambandam S (2023). Total hip arthroplasty in cirrhosis is associated with increased complications during the hospital stay, length of stay, and cost of care: a propensity matched database study. Arch Bone Jt Surg.

[24] Koh A, Bull N, Brown L, Thomson B, Loveday BP (2024). Prospective cohort study of rotational thromboelastometry in established biliary obstruction: dispelling the myth of auto-anticoagulation. HPB (Oxford).

[25] Eberhard CM, Davis KA, Nisly SA (2022). Adherence to goal-directed therapy for patients with cirrhosis hospitalized with spontaneous bacterial peritonitis. Am J Health Syst Pharm.

[26] Wong NZ, Mahmud N (2020). The imperative for an updated cirrhosis surgical risk score. Ann Hepatol.

[27] Li YT, Wang YC, Yang SF (2022). Risk factors and prognoses of invasive Candida infection in surgical critical ill patients with perforated peptic ulcer. J Microbiol Immunol Infect.

